# Association of electronic cigarette use with self-reported difficulty concentrating, remembering, or making decisions in US youth

**DOI:** 10.18332/tid/130925

**Published:** 2020-12-22

**Authors:** Catherine Xie, Zidian Xie, Dongmei Li

**Affiliations:** 1 Pittsford Sutherland High School, Pittsford, United States; 2 University of Rochester Clinical and Translational Science Institute, University of Rochester Medical Center, Rochester, United States

**Keywords:** vaping, smoking, cognitive complaints, youth

## Abstract

**INTRODUCTION:**

Electronic cigarette use (vaping) has become increasingly popular among youth. The aim of this study is to determine the cross-sectional association of vaping, smoking, and dual use of these tobacco products with self-reported serious difficulty concentrating, remembering, or making decisions (DCRMD), because of a physical, mental, or emotional condition (PMEC) in US youth.

**METHODS:**

The 2018 National Youth Tobacco Survey (NYTS) data with 18535 youth were used for analysis. All included youth who answered whether they have serious DCRMD and stated their vaping and smoking status. Multivariable weighted logistics regression models were used to examine the association of vaping and smoking with the risk of DCRMD in youth, considering a complex sampling design.

**RESULTS:**

Ever dual users (AOR=4.19; 95% CI: 2.97–5.92), exclusive ever cigarette smokers (AOR=1.50; 95% CI: 1.18–1.91) and exclusive ever e-cigarette users (AOR=3.13; 95% CI: 2.25–4.34) had significantly higher odds of self-reported DCRMD than never users in youth. Subgroup analysis on exclusive ever e-cigarette users who started vaping in middle school or earlier had significantly higher odds of self-reported DCRMD compared to those who started vaping in high school (AOR=1.77; 95% CI: 1.27–2.45). Meanwhile, male youth who were exclusive ever e-cigarette users had higher odds of self-reported DCRMD than female youth who were exclusive ever e-cigarette users (AOR=1.67; 95% CI: 1.25–2.22).

**CONCLUSIONS:**

Vaping, smoking and dual use were associated with self-reported serious difficulty concentrating, remembering, or making decisions because of a physical, mental, or emotional condition in youth, which provided initial evidence on the cross-sectional association between vaping and self-reported cognitive problems.

## INTRODUCTION

During the past few years, e-cigarette use has increased substantially and e-cigarettes have become the most widely used tobacco product among youth^1^. In 2019, about 27.5% of high school students and 10.5% of middle school students reported use of e-cigarettes in the US, which suggests that around 4.1 million high school students and 1.2 million middle school students have used e-cigarettes in 2019^1^. This is a dramatic increase from 2017 when an estimated 11.7% of high school students used e-cigarettes^2^. The increasing popularity of e-cigarette use in youth raises serious public health concerns since recent studies showed the association of e-cigarette use with many health conditions and symptoms, such as respiratory diseases and heart problems^3,4^. Mice studies have indicated that e-cigarette use is associated with cardiovascular diseases and cancer^5,6^. Previous human studies have shown that e-cigarette use is associated with respiratory symptoms and asthma in youth^7,8^. Recent combined 2016 and 2017 national Behavioral Risk Factor Surveillance System (BRFSS) survey data analysis showed a significant association of e-cigarette use with cognitive complaints in US adults^9^.

Maternal smoking has been found to be associated with increased risk of cognitive and auditory deficits in offspring^10^. Adolescence is a period of critical brain development for mature cognitive functions and working memory, during which the developmental brain is vulnerable to nicotine, tobacco, and e-cigarettes^11,12^. Previous study has found that long-term smoking could lead to DCRMD^13^. Other studies showed that adolescent smokers had lower scores than age-matched non-smokers when given tests of verbal comprehension, oral arithmetic, memory, and auditory memory^14,15^. A twin study indicated that adolescent smoking leads to attention problems that can last through adulthood^16^. All this evidence indicates that smoking is highly associated with DCRMD in youth.

An animal study suggested that nicotine is behind the relationship between smoking and attention problems^16^. While acute nicotine exposure could impact brain development and make adolescents susceptible to future substance abuse, chronic nicotine exposure could alter cognitive functions^11,12^. Animal research provides evidence that nicotine disrupts adolescent brain development, changing cell signaling and cholinergic neurotransmission^10^. Previous human studies have found that youth brain development could be harmed and their learning, memory and attention could be affected by nicotine exposure during adolescence^17,18^. During adolescence, myelination (which allows faster electrical and neural signaling) occurs in the frontal lobe of the brain that controls reasoning, decision-making skills, self-discipline, and impulse control^19^. Any interference in myelination due to nicotine, can cause mild to severe cognitive and learning deficits^19^. Therefore, it is reasonable to speculate that adolescents are more sensitive to nicotine than adults.

Since both e-cigarettes and cigarettes contain nicotine, it is possible that cognition functions could also be affected by e-cigarette use, especially among youth. However, no previous study has shown a link between e-cigarette use and cognitive functions in youth, which is an important public health issue that needs to be further explored. We hypothesize that e-cigarette use is associated with serious DCRMD because of a PMEC in youth. Using the 2018 National Youth Tobacco Survey data on middle school and high school students, we aim to investigate the cross-sectional association of e-cigarette use with serious self-reported DCRMD because of a PMEC in adolescents.

## METHODS

### Study participants

The National Youth Tobacco Survey (NYTS) is an annual cross-sectional national survey administered to US high school and middle school students^20^. The survey has been conducted with pencil and paper since 1999, asking about tobacco-related beliefs, attitudes, behavior, and exposure to tobacco influences. A stratified, three-stage cluster sampling design generates a nationally representative sample of all US students from grade 6 to 12. We used the 2018 NYTS data that included 20189 participants, a combination of middle and high school students. The 2018 NYTS data can be publicly accessed from the Center for Disease Control and Prevention website (https://www.cdc.gov/tobacco/data_statistics/surveys/nyts/data/index.html).

### Outcome variable and covariates

One of the survey questions was: ‘Because of a physical, mental, or emotional condition, do you have serious difficulty concentrating, remembering, or making decisions?’, which was the self-reported cognitive outcome variable in this study. Only the students who answered ‘yes’ or ‘no’ to this question were included in the data analysis. The predictor variable was created by combining the ever cigarette smoking variable and ever e-cigarette use variable, with answers of ‘yes’ or ‘no’ to the questions: ‘Have you ever tried cigarette smoking, even one or two puffs?’ and ‘Have you ever used an e-cigarette, even once or twice?’. The predictor variable of smoking and vaping status had four categories: 1) ever dual users (‘yes’ answers to both the ever cigarette smoking and ever e-cigarette use questions); 2) exclusive ever cigarette smokers (‘yes’ answer to the ever cigarette smoking question and ‘no’ answer to the ever e-cigarette use question); 3) exclusive ever e-cigarette users (‘no’ answer to the ever cigarette smoking question and ‘yes’ answer to the ever e-cigarette use question); and 4) never users (‘no’ answers to both the ever cigarette smoking and ever e-cigarette use questions). The covariates controlled for in the multivariable weighted logistic regression analysis were: age; gender; race; other tobacco use (such as use of roll-your-own cigarettes; use of tobacco in hookah; use of snus, use of chewing tobacco, snuff, or dip, such as Redman, Levi Garrett, Beechnut, Skoal, Skoal Bandits, or Copenhagen); during the past 30 days had a strong craving or felt like you really needed to use a tobacco product of any kind; number of days (0, 1–2, 3–5, 6–9, 10–19, 20–29, 30 days) of any tobacco product use (including cigarettes, cigars, smokeless tobacco, e-cigarettes, hookahs, pipes, snus, dissolvable tobacco, and bidis) during the past 30 days; whether smoked cigarettes at least one day in past 30 days; and age of first use of e-cigarettes. The subgroup analysis on exclusive ever e-cigarette users controlled for the same covariates except for whether smoked cigarettes at least one day in past 30 days.

### Statistical analysis

Weighted frequency distributions and summary statistics were used to examine the unadjusted association between predictor variable or each covariate and the risk of serious DCRMD because of a PMEC to identify potential confounding variables. Weighted multivariable logistic regression models were used to determine the association of vaping, smoking, and dual use with self-reported serious DCRMD because of a PMEC in youth, after controlling for the covariates. The stratification variable *stratum2*, clustering variable *psu2*, and final weight variable *finwgt* were used in the model to account for the complex sampling design of the NYTS data. Adjusted odds ratios (AORs) and their 95% confidence intervals were used to examine the association between e-cigarette use and self-reported serious DCRMD because of a PMEC in youth. Purposeful model selection method was used to select significant covariates in the final weighted multivariable logistic regression model^21^.

Multicollinearities among covariates were examined using the variance inflation factor (VIF) values to ensure that no multicollinearities existed among the covariates. All analyses were conducted using the *proc survey* procedures in SAS V 9.4 (SAS Institute Inc., Cary, NC). All tests were two-sided with a significance level of 5%.

## RESULTS

### Demographic characteristics of youth who self-reported DCRMD

Out of the 20189 participants, we included 18535 youth who gave an answer about their vaping/smoking status and self-reported serious DCRMD because of a physical, mental, or emotional condition. Among the 18535 youth, there were 2354 youth who were ever dual users (12.70%), 861 youth who were exclusive ever cigarette smokers (4.65%), 2386 youth who were exclusive ever e-cigarette users (12.87%), and 12934 youth who were never users (69.78%). Overall, there were 3285 out of 18535 youth who self-reported serious DCRMD because of a PMEC (17.68%). Table 1 shows that the occurrence of self-reported serious DCRMD because of a PMEC was the highest in ever dual users (31.01%), followed by exclusive ever cigarette smokers (24.92%), exclusive ever e-cigarette users (19.69%), and never users (14.53%). The prevalence of self-reported serious DCRMD because of a PMEC was higher in males (19.94%) than females (15.63%). Non-Hispanic American Indians and Alaska Natives (NH-AI/AN) reported the highest prevalence of serious DCRMD because of a PMEC (30.73%). Youth who reported other tobacco use had higher prevalence of self-reported serious DCRMD because of a PMEC (26.99%) than youth who did not use other tobacco products (15.50%). Youth who had a strong craving for tobacco products had a higher percentage of self-reported serious DCRMD because of a PMEC than youth who did not (42.04% vs 16.07%). Youth who used any tobacco product in past 30 days had a higher proportion of self-reported serious DCRMD because of a PMEC than youth who did not use any tobacco product in past 30 days. Youth who reported having smoked at least one day in the past 30 days had a higher prevalence of self-reported serious DCRMD because of a PMEC than youth who did not (39.08% vs 16.43%). The mean age (years) of youth was similar between youth who self-reported serious DCRMD because of a PMEC (mean=14.7; 95% CI: 14.5–14.8) and youth who did not report serious DCRMD because of a PMEC (mean=14.6; 95% CI: 14.4–14.8). The mean age of first e-cigarette use was significantly older in youth who self-reported serious DCRMD because of a PMEC (mean=9.3; 95% CI: 9.1–9.6) than youth who had not self-reported serious DCRMD because of a PMEC (mean=8.7; 95% CI: 8.5–8.8).

**Table 1:** Frequency and weighted percentage of self-reported serious difficulty concentrating, remembering, or making decisions (DCRMD) because of a physical, mental, or emotional condition (PMEC), on each variable

*Variable*		*Self-reported serious DCRMD*		
	*n*	*Yes (n=3285) Weighted % (95% CI)*	*No (n=15250) Weighted % (95% CI)*	*p*
**Smoking and vaping status**				<0.0001
Ever dual users	2354	31.01 (29.83–31.87)	68.99 (69.05–68.94)	
Exclusive ever cigarette smokers	861	24.92 (22.76–26.51)	75.08 (74.64–75.40)	
Exclusive ever e-cigarette users	2386	19.69 (19.35–19.95)	80.31 (79.46–80.92)	
Never users	12934	14.53 (14.32–14.70)	85.47 (85.16–85.70)	
**Sex**				<0.0001
Male	9526	19.94 (19.47–20.31)	80.06 (79.93–80.17)	
Female	9426	15.63 (15.33–15.87)	84.37 (84.10–84.57)	
**Race**				0.0012
NH-White	8638	16.65 (16.24–16.95)	83.35 (82.91–83.67)	
NH-Black	2217	18.42 (17.63–18.94)	81.58 (81.72–81.48)	
Hispanic	5448	20.49 (20.21–20.69)	79.51 (79.32–79.66)	
NH-Asian	703	11.22 (11.15–11.25)	88.78 (86.75–89.77)	
NH-AI/AN	278	30.73 (27.59–32.33)	69.27 (66.70–70.58)	
NH-NHOPI	95	22.05 (17.82–23.76)	77.95 (72.75–80.06)	
Multiple races	968	20.94 (19.47–21.99)	79.06 (78.66–79.35)	
**Other tobacco use**				<0.001
Yes	4044	26.99 (26.34–27.48)	73.01 (72.61–73.31)	
No	15053	15.50 (15.21–15.73)	84.5 (84.33–84.64)	
**During the past 30 days, have you had a strong craving or felt like you really needed to use a tobacco product of any kind?**				<0.0001
Yes	1308	42.04 (41.03–42.78)	57.96 (56.81–58.82)	
No	17615	16.07 (15.76–16.32)	83.93 (83.76–84.07)	
**During the past 30 days, on how many days did you use any tobacco product?**				<0.0001
0	16575	16.37 (16.07–16.61)	83.63 (83.44–83.78)	
1–2	691	25.58 (22.97–27.42)	74.42 (73.04–75.39)	
3–5	347	28.74 (23.73–32.23)	71.26 (71.41–71.16)	
6–9	255	22.23 (20.25–23.42)	77.77 (75.68–79.02)	
10–19	254	35.87 (32.91–37.81)	64.13 (62.59–65.14)	
20–29	232	22.32 (18.15–24.88)	77.68 (75.64–78.94)	
30	549	33.60 (32.36–34.40)	66.40 (64.05–67.94)	
**Smoked cigarettes on ≥1 of the past 30 days**				<0.0001
Yes	1000	39.08 (38.20–39.69)	60.92 (59.57–61.85)	
No	17700	16.43 (16.17–16.63)	83.57 (83.38–83.72)	

NH: Non-Hispanic. AI/AN: American Indians/Alaska Natives. NHOPI: Native Hawaiian or Other Pacific Islander. CI: confidence interval.

### Association of smoking and vaping status with self-reported serious DCRMD because of a PMEC

**Figure 1:**
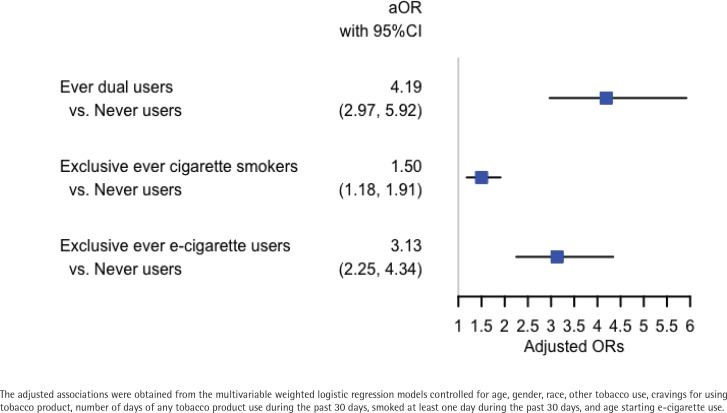
Association of smoking and vaping status with self-reported serious difficulty of concentrating, remembering, or making decisions because of a physical, mental, or emotional condition in youth

Using univariate and multivariable weighted logistic regression models, we calculated the unadjusted and adjusted odds ratios (AORs) of having self-reported serious DCRMD because of a PMEC. Figure 1 shows the adjusted odds ratios of serious DCRMD because of a PMEC when comparing ever dual users, exclusive ever cigarette smokers, exclusive ever e-cigarette users with never users. Ever dual users had the highest association with self-reported serious DCRMD because of a PMEC (AOR=4.19; 95% CI: 2.97–5.92). Exclusive ever e-cigarette users (AOR=3.13; 95% CI: 2.25–4.34) and exclusive ever cigarette smokers (AOR=1.50; 95% CI: 1.18–1.91) had a significantly higher association with self-reported serious DCRMD because of a PMEC than never users. Together, vaping and smoking had similar association with self-reported serious DCRMD because of a PMEC. The adjusted odds ratios of covariates and their 95% confidence intervals are given in the Supplementary file (Table S1). We note that both gender and the age of first e-cigarette use have a significant adjusted associations with self-reported serious DCRMD because of a PMEC in youth.

### Association of gender and age of first e-cigarette use with self-reported serious DCRMD because of a PMEC

**Figure 2:**
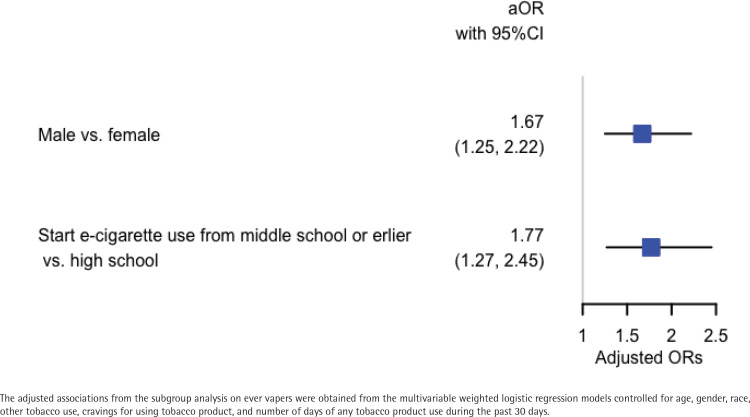
Association of gender and the age of starting vaping with self-reported serious difficulty of concentrating, remembering, or making decisions because of a physical, mental, or emotional condition in youth who ever vaped

To further examine whether similar associations of gender and age of first e-cigarette use with self-reported serious DCRMD because of a PMEC existed in exclusive ever e-cigarette users, we conducted a subgroup analysis for only exclusive ever e-cigarette users. There were 1218 males (50.99%) and 1203 females (49.01%) in the 2421 exclusive ever e-cigarette users who reported their genders. Among the 2431 exclusive ever e-cigarette users who reported their age of first e-cigarette use, 745 youth (29.02%) reported first e-cigarette use between 8 and 13 years old. The remaining 1686 (70.98%) youth reported first e-cigarette use when at least 14 years old. Figure 2 shows that among exclusive ever e-cigarette users, males had significantly higher odds of self-reported serious DCRMD because of a PMEC than females (AOR=1.67; 95% CI: 1.25–2.22). Meanwhile, starting using e-cigarettes in middle school or earlier had significantly higher odds of self-reported serious DCRMD because of a PMEC compared to starting using e-cigarettes in high school (AOR=1.77; 95% CI: 1.27–2.45).

## DISCUSSION

Previous studies on nicotine and difficulty concentrating, remembering, or making decisions (such as attention deficit hyperactivity disorder) primarily focused on prenatal and adolescent exposure to smoking^10,22^. Using the 2018 National Youth Tobacco Survey, we investigated the cross-sectional association (directly or indirectly) of smoking and vaping with self-reported serious DCRMD because of a PMEC in youth. Consistent with previous findings, our study showed that smokers had a significantly higher association with self-reported serious DCRMD because of a PMEC compared to never users in youth^16^. More importantly, we found youth who ever vaped (either exclusive vapers or dual users) had a significantly higher association with self-reported serious DCRMD because of a PMEC than students who never vaped and smoked.

During adolescence, the brain is still developing and vulnerable to neurotoxicants like nicotine^23^. Previous studies on cognitive effects of nicotine have found that early exposure to nicotine could impact the brain development in youth and lead to cognitive deficits in later life^16,24^. A study conducted on young adults aged 18–29 years found smokers had significant cognitive impairment on sustained attention and spatial working memory compared to non-smokers^25^. Our study showed that vaping had a similar association with self-reported serious DCRMD because of a PMEC in youth as smoking. As e-cigarettes also contain nicotine, it is plausible that similar associations exist between vaping and cognitive problems as smoking, evidenced from our current study. In addition, it has been reported that some e-cigarettes could contain and generate similar amounts of nicotine compared to cigarettes^26^. Previous study also showed that the amount of nicotine delivered through e-cigarettes could exceed that of combustible cigarettes^27^. This provides one possible explanation for the similar association with self-reported serious DCRMD because of a PMEC between vaping and smoking.

Ever use of e-cigarettes, even once or twice, together with ever smoked even one or two puffs, were used to create the composite exposure variable to examine the association of dual use, exclusive e-cigarette use, and exclusive smoking, with the cognitive problems in youth. Ever use of e-cigarette was chosen due to previous evidence on the association of ever e-cigarette use with health conditions^28,29^. A longitudinal analysis using the nationally representative Population Assessment of Tobacco and Health (PATH) Waves 1, 2, and 3 adult data showed significant associations of both former e-cigarette use (including even once or twice) and current e-cigarette use at Wave 1 with incident respiratory disease at Waves 2 or 3^29^. Another study using the combined 2016 and 2017 BRFSS survey data showed that former e-cigarette use (including even just one-time use) had a significant association with a history of clinical diagnosis of depression^28^. Thus, ever use of e-cigarettes, which includes both former use and current use of e-cigarettes, was used as the exposure variable to examine its association with the cognitive problems in youth.

Our subgroup analysis showed that youth who started using e-cigarettes in middle school or earlier had significantly higher odds of self-reported serious DCRMD because of a PMEC than youth who started using e-cigarettes in high school. This indicates the timing for future prevention interventions to mitigate tobacco use among youth. An intervention program for reducing tobacco use in youth could be more effective if the program starts with middle school or even elementary school students. Previous study found that boys were more likely than girls to be affected by cognitive developmental disorders during their childhood^30^. Current subgroup analysis also observed higher odds of serious DCRMD because of a PMEC in male youth who were exclusive ever e-cigarette users than in female youth who were exclusive ever e-cigarette users. Previous studies on gender differences in cigarette smoking have found that females could metabolize nicotine and cotinine faster than males and are less sensitive to the rewarding of nicotine, which might make females harder to quit smoking than males^31-35^. How vaping affects youth and adult males and females differently is currently unknown, which needs further investigation.

### Limitations

There are limitations in the present study. First, this cross-sectional national survey study only investigated the association between smoking/vaping and selfreported serious DCRMD because of a PMEC in youth, and does not imply any causal relationship between vaping and self-reported DCRMD in youth. It is possible that youth who vaped might have higher risk for a PMEC that led to a serious DCRMD. Alternatively, it is also plausible that youth who had cognitive problems might try to use vaping or smoking to attenuate their PMEC symptoms. Future longitudinal studies should be conducted to explore the possible causal associations between vaping and serious DCRMD in youth. Second, the exposure variable of ever use of e-cigarettes lacks information on lifetime vaping occurrence. The association of one or two times e-cigarette use with DCRMD because of a PMEC could be different from the association from more frequent e-cigarette use. Unfortunately, the 2018 National Youth Tobacco Survey data did not include lifetime occurrence information. Future research is needed to examine the relationship of e-cigarette use extent and cognitive problems. Third, in our current research the definition of the outcome variable refers to serious DCRMD because of PMEC. Thus, the association of ever e-cigarette use with cognitive problems might not be direct. The PMEC could be a mediator between ever e-cigarette use and cognitive problems. It is plausible that ever e-cigarette use is associated with a PMEC that caused the serious DCRMD. Direct associations between e-cigarette use and cognitive problems need to be explored in future studies. Fourth, some potential confounding variables such as the lifestyle and health status of the youth were not adjusted in the analysis due to lack of information in the NYTS questionnaire. Finally, like all other survey studies, the National Youth Tobacco Survey might contain some recall bias. Self-reported smoking and vaping status as well as self-reported serious DCRMD because of a PMEC might be unreliable, which might lead to underestimation or overestimation in the analysis results. Given the short time period of e-cigarettes in the market, it is hard to determine the long-term effects of e-cigarette use. Therefore, more data need to be collected and analyzed in the future to evaluate the long-term cognitive effects of vaping in youth.

## CONCLUSIONS

This cross-sectional study showed an association of smoking/vaping with self-reported serious difficulty concentrating, remembering, or making decisions (DCRMD) because of a physical, mental, or emotional condition (PMEC) in youth. Results from the subgroup analysis showed that the younger an adolescent started vaping, the higher their association with self-reported serious DCRMD because of a PMEC. Further studies are warranted to investigate the effect of vaping on cognitive problems in youth.
