# Supplementary material for: Association of electronic cigarette use with self-reported difficulty concentrating, remembering, or making decisions in US youth
Source: Tob Induc Dis. 2020 Dec 22;18:106. doi: 10.18332/tid/130925 (PMC7759092; doi:10.18332/tid/130925)
Supplement: Supplementary file 1 [file TID-18-106-s1.pdf]

**Supplemental Table S1: Adjusted odds ratios of self-reported difficulty concentrating, remembering, or making decisions because of a physical, mental, or emotional condition in youth.**

| **Variables** | **Adjusted Odds Ratio (95% CI)** |
|---|---|
| Smoking and Vaping Status | |
| Ever dual users | 4.19(2.97,5.92) |
| Exclusive ever cigarette smokers | 1.50(1.18,1.91) |
| Exclusive ever e-cigarette users | 3.13(2.25,4.34) |
| Never users | Reference |
| Age of participant | 0.98(0.95,1.01) |
| Sex | |
| Male | 1.40(1.27,1.54) |
| Female | Reference |
| Race/Ethnicity | |
| Non-Hispanic Black | 1.19(1.00,1.42) |
| Hispanic | 1.29(1.12,1.48) |
| Non-Hispanic Asian | 0.70(0.50,0.98) |
| Non-Hispanic AI/AN | 1.93(1.36,2.73) |
| Non-Hispanic NHOPI | 1.44(0.79,2.62) |
| Multiple races | 1.26(0.99,1.61) |
| Non-Hispanic White | Reference |
| Other tobacco use | |
| Yes | 1.07(0.91,1.27) |
| No | Reference |
| During the past 30 days, have you had a strong craving or felt like you really needed to use a tobacco product of any kind? | |
| Yes | 2.64(2.14,3.24) |
| No | Reference |
| During the past 30 days, on how many days did you use any tobacco product(s)? | |
| 1 or 2 days | 1.01(0.75,1.37) |
| 3 to 5 days | 1.07(0.74,1.55) |
| 6 to 9 days | 0.50(0.34,0.74) |
| 10 to 19 days | 1.13(0.74,1.73) |
| 20 to 29 days | 0.45(0.26,0.79) |
| All 30 days | 0.64(0.44,0.93) |
| 0 days | Reference |
| Reported you smoked cigarettes on >= 1 of the past 30 days | |
| Yes | 1.52(1.17,1.98) |
| No | Reference |
| How old were you when you first tried using an e-cigarette, even once or twice? | 0.89(0.85,0.93) |

© 2020 Xie C. et al.
